# Ecdysone signaling acts as a regulator of immunity and microbiota balance, shaping *Leishmania infantum* infection in *Phlebotomus perniciosus*

**DOI:** 10.3389/fimmu.2026.1904500

**Published:** 2026-08-11

**Authors:** Cecilia Stahl Vieira, Marco Salvemini, Petr Volf, Erich Loza Telleria

**Affiliations:** 1 Department of Parasitology, Faculty of Science, Charles University, Prague, Czechia; 2 Department of General Biology, Institute of Biology, Fluminense Federal University, Niterói, Rio de Janeiro, Brazil; 3 Department of Biology, University of Naples Federico II, Naples, Italy

**Keywords:** antimicrobial peptides (AMPs), ecdysone signaling, immune signaling pathways, *Leishmania infantum*, microbiota, *Phlebotomus perniciosus*, RNA interference (gene silencing), sand flies

## Abstract

**Background:**

The steroid hormone ecdysone, acting through its nuclear receptor (EcR), is a key regulator of insect development and reproduction and also contributes to immune regulation and gut microbiota homeostasis. In sand flies, both immunity and the gut microbiota are critical determinants for *Leishmania* establishment in the midgut. *Phlebotomus perniciosus* is the principal vector of *Leishmania infantum* in the western Mediterranean basin; however, the mechanisms affecting interactions among parasite, vector immune system, and resident microbiota remain poorly understood in this species.

**Methods:**

Using RNA interference–mediated silencing of EcR, we assessed how disruption of ecdysone signaling affects vector immunity, gut bacteria, and parasite development. Double-stranded RNA targeting EcR or a control sequence was microinjected into *L. infantum*–infected females, and gene expression was analyzed by RT-qPCR. Parasite load was quantified via *Leishmania* actin expression, while gut bacterial relative abundance was assessed using universal 16S rRNA primers.

**Results:**

*L. infantum* infection induced expression of the sand fly ecdysone signaling–related genes, immune pathway transcription factors, and antimicrobial peptides (AMPs), while significantly reducing gut bacterial load during the late phase of parasite infection. In contrast, EcR silencing suppressed downstream ecdysone-related genes, impaired activation of the immune deficiency (Imd) and JAK/STAT pathways, and disrupted AMPs expression. This was associated with a marked increase in bacterial relative abundance in sugar-fed sand flies and altered microbial and parasite loads in *Leishmania*-infected sand flies.

**Discussion:**

Collectively, our results indicate that ecdysone signaling regulates the Imd and JAK/STAT transcription factors, thereby modulating *P. perniciosus* immune responses and suppressing the gut microbiota. Disruption of ecdysone signaling reveals an inverse correlation between parasite burden and bacterial abundance.

**Conclusion:**

These findings identify ecdysone signaling as a central regulator coordinating immune responses and maintaining the balance between *Leishmania* and the gut microbiota in sand flies.

## Graphical Abstract

**Figure:**
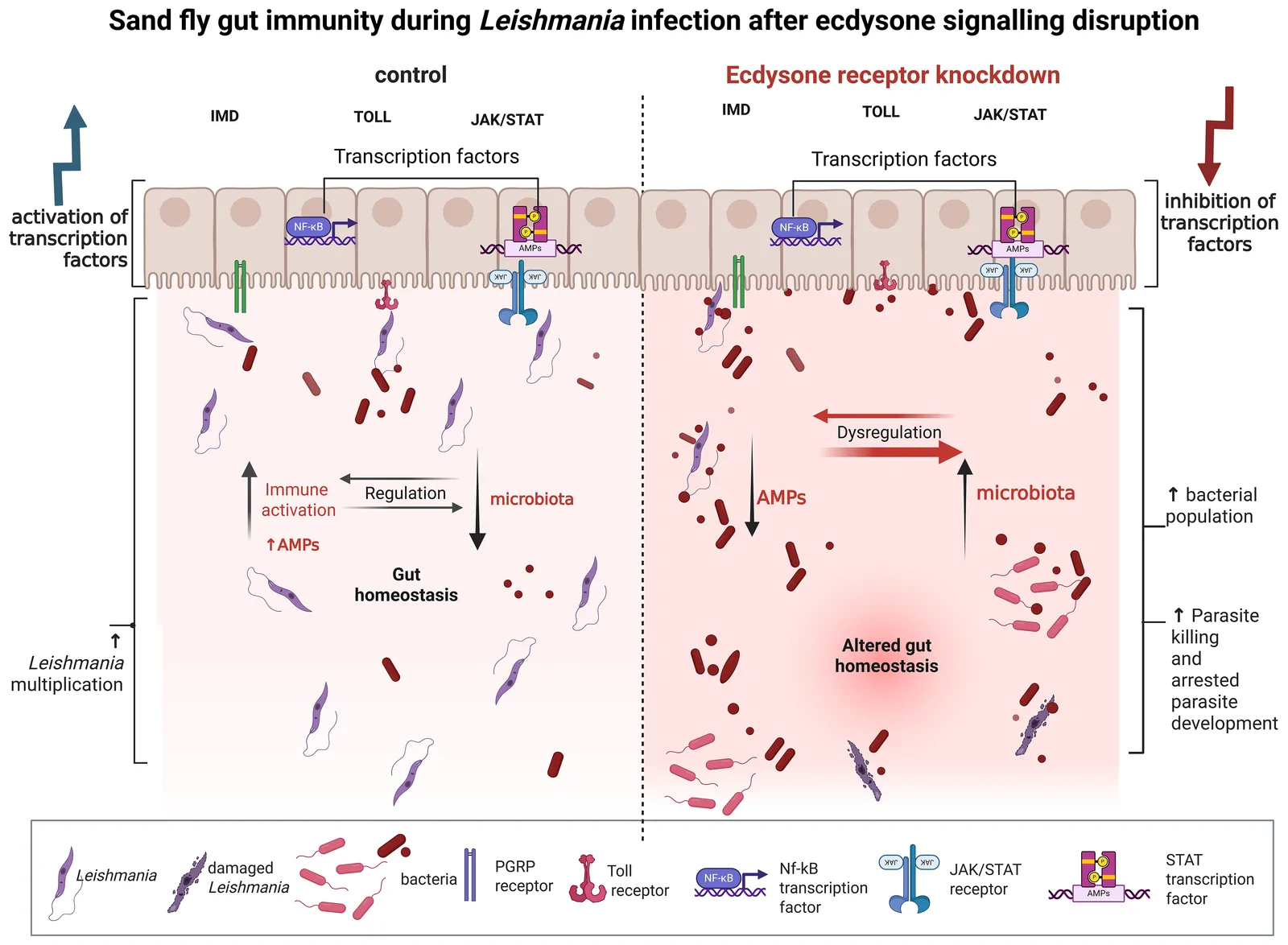
Created in BioRender. Stahl vieira, C. (2026) https://BioRender.com/.

## 1 Introduction

Ecdysone is a steroid hormone that exerts temporal and tissue-specific regulation over fundamental physiological processes in insects, including molting, metamorphosis, and reproduction (1, 2). It is synthesized and secreted by the prothoracic glands in its active form, 20-hydroxyecdysone (20E), which is released into the hemolymph. In target cells, 20E binds to a nuclear heterodimer receptor composed of the ecdysone receptor (EcR) and the ultraspiracle protein (USP) (3, 4). EcR is broadly expressed across diverse tissues and cell types, enabling systemic hormonal responses (5). Upon activation, the EcR/USP complex binds to specific ecdysone-responsive motifs in regulatory regions of target genes (6, 7), initiating the transcription of early and late response genes that orchestrate insect development and reproduction (4, 8). These hierarchical transcriptional events highlight the ecdysone pathway as an integrator between systemic hormonal cues and gene regulation.

Beyond its classical role in development and reproduction, ecdysone signaling in *Drosophila melanogaster* also affects immunity. It modulates the expression of immune effectors such as antimicrobial peptides (AMPs) (9, 10), and regulates immune homeostasis during tissue remodeling (11) and infection (12). In addition, ecdysone signaling modulates transcription of key components of the immune deficiency (Imd) pathway, such as the pattern recognition receptor PGRP-LC, as well as AMPs, through mechanisms that can be either dependent on or independent of microbial stimulation (12, 13). Collectively, ecdysone acts as a hormonal regulator that interfaces developmental and physiological status with innate immune responsiveness.

The disruption of ecdysone signaling in insect vectors impairs Imd-related immune responses and alters susceptibility to pathogens. In *Anopheles gambiae*, 20E promotes the expression of immune genes that affect *Plasmodium* development, reducing oocyst load and parasite survival (14, 15). Similarly, EcR silencing in *Anopheles funestus* impairs reproductive fitness and significantly reduces *Plasmodium falciparum* infection intensity (16). In *Rhodnius prolixus*, azadirachtin-induced inhibition of ecdysone secretion suppresses relish and AMP expression, thereby disturbing midgut immune homeostasis (17). Interestingly, in *Aedes aegypti*, EcR silencing enhances Imd pathway activation and bacterial resistance, though at the cost of reduced fertility (18). These studies underscore a conserved role for ecdysone signaling in mediating physiological trade-offs between development, immunity, and vector competence across diverse hematophagous insects, yet its role in sand fly immunity remains poorly defined.

Using the sand fly *Phlebotomus perniciosus*, the main vector of *Leishmania infantum* in the Western Mediterranean basin (19, 20), our group provided the first experimental evidence of neuroendocrine–immune crosstalk in sand flies. Previously, we showed that chemical inhibition of ecdysone synthesis by azadirachtin leads to transcriptional suppression of ecdysone-responsive genes and AMPs in whole bodies of larvae and females. Analogously, this transcriptional impairment was reversed by exogenous 20E supplementation, indicating that ecdysone signaling directly influences immune competence in sand flies (21). Considering that the Imd pathway and its downstream transcription factor relish are essential in sand flies for maintaining gut microbiota homeostasis and controlling *Leishmania* infection (22–24), we hypothesized that hormonal pathways could shape parasite-vector interactions through immune regulation. Given that sand fly vector competence depends on a finely tuned balance between immune activation and gut microbial homeostasis, endocrine regulation may represent a central coordinating mechanism linking gut physiology to the establishment of *Leishmania* infection.

Here, we investigated whether ecdysone signaling modulates immune responses in the sand fly gut and influences *P. perniciosus* susceptibility to *L. infantum*, by silencing the *EcR* gene via RNA interference (RNAi) in adult females. The effects of *EcR* knockdown were evaluated by measuring the expression of EcR, the transcriptional repressor Eip75B, and the GATA transcription factor serpent, representing key regulators of the ecdysone signaling pathway. In parallel, we assessed the expression of transcription factors relish, dorsal, and STAT belonging to three main immunity pathways Imd, Toll, and JAK/STAT, respectively. We also investigated the expression of three AMPs (*defensin 1*, *defensin 2*, and *attacin*). These selected genes represent core nodes of the ecdysone cascade, major innate immune pathways, and key effector outputs, providing a focused panel that links hormonal signaling, immune regulation, and downstream antimicrobial activity. In addition, we assessed *Leishmania* development in the sand fly midgut and the potential consequences of hormonal disruption on gut microbiota to provide a broader understanding of endocrine–immune–parasite interactions relevant to sand fly vector competence.

## 2 Methods

### 2.1 Leishmania infantum culture

*Leishmania infantum* (MHOM/TR/2000/OG-VL) promastigotes were maintained at 23 °C and cultured in Medium 199 (Sigma-Aldrich, Darmstadt, Germany) supplemented with 10% heat-inactivated fetal bovine serum (Thermo Fisher Scientific, Carlsbad, CA, United States), 1% BME vitamins (Sigma-Aldrich), and 250 µg/mL amikacin (Medopharm, Pozořice, Czech Republic).

### 2.2 Phlebotomus perniciosus colony

*Phlebotomus perniciosus* females, originally captured in Murcia, Spain, were maintained for more than 15 years under standardized conditions at the Department of Parasitology, Faculty of Science, Charles University in Prague, Czech Republic, following previously described protocols (25). Prior to each experiment, approximately 300 recently emerged *P. perniciosus* females were randomly selected and maintained without sugar for 24 hours. Depending on the experimental design, sand flies were then fed on sugar *ad libitum*, blood, or blood seeded with *L. infantum*.

### 2.3 Sand fly blood feeding and artificial infection

For blood feeding and *L. infantum* infection, defibrinated sheep blood (LabMedia, Jaroměř, Czech Republic) was heat-inactivated at 56 °C for 35 minutes and then cooled to room temperature before feeding. For the infection, the inactivated blood was seeded with *L. infantum* (1 × 10^6^ promastigotes/mL of blood), as detailed in previous reports (26, 27). Control blood or infectious blood meals were offered on an artificial glass feeder covered with a chicken skin membrane. One day after feeding, fully engorged females were separated, and guts were dissected in sterile saline solution on the following days (1, 2, 3, 5, 7, and 9), depending on each experimental design. Samples were collected in three pools of five guts for each time point and stored at −80 °C until RNA extraction. A minimum of three independent experiments was performed.

### 2.4 Double-strand RNA synthesis and injection in *P. perniciosus* females

Templates for double-stranded RNA (dsRNA) synthesis were amplified by PCR using *P. perniciosus* cDNA and gene-specific primers for EcR gene. The control dsRNA template was amplified from the p-GEM-T Easy plasmid (Promega, Madison, WI, United States) using *Escherichia coli* beta-galactosidase (LacZ) primers. Both sets of primers for EcR or LacZ dsRNA templates contain the T7 promoter sequence at the 5’end (**Table 1**). The PCR cycling conditions were as follows: 95 °C for 3 min; 34 amplification cycles (95 °C for 30 s; 60 °C for 30 s, 72 °C for 1 min); and 72 °C for 5 min. Amplicons were visualized on 1% agarose gel and purified using a DNA Gel Extraction kit (Roche, Pleasanton, CA, USA). Purified templates were used in dsRNA synthesis using the MEGAscript RNAi Kit (Invitrogen, Carlsbad, CA, United States) according to the manufacturer’s instructions. EcR- or LacZ-specific dsRNA (dsEcR or dsLacZ, respectively) was lyophilized and resuspended in sterile H_2_O to a final concentration of 4.5 μg/μL. For gene silencing, 64 nL of dsEcR of dsLacZ were microinjected into the thorax of sand fly females anesthetized on ice using Nanoject II microinjector (Drummond, Broomall, PA, United States) (24, 32). Sand flies used in dsRNA injections were either sugar-fed, blood-fed, or *Leishmania*-infected, depending on the experimental design.

**Table 1:** Primer sequences used for dsRNA synthesis and RT-qPCR analyses.

Name	Sequence
dsEcR (present study)	* TAATACGACTCACTATAGGGAGACTACGATCCCTACAGTCCCAATG
	* TAATACGACTCACTATAGGGAGACTGTAATGTGTCGGAAGTGAACG
dsLacZ adapted from (28)	* TAATACGACTCACTATAGGGAGATATCCGCTCACAATTCCACA
	* TAATACGACTCACTATAGGGAGAGAGTCAGTGAGCGAGGAAGC
GAPDH (21)	AGTCCACGGGAGTGTTTACCA
	CGCCGCCCTTGAGATG
EF-1α (29)	GTGTCATCAAGGCTGTCAACTTC
	TGGCTAGCTACTTCTTGGTCTTG
EcR (21)	TGTCAAAGTAGAGCCAATACACG
	GATGACAACCAATCGTCACCTTC
Eip75B (21)	ATGTGGTCGATGGAGGAGGA
	CTGGAGACTGAGCCCATTGG
serpent (21)	CTCGGCGAACCAGTGTGTAA
	TGGCTTCCTTTTGCGAGTCT
relish (present study)	ATCTCGCTTGTGCTCTGGAC
	CGACATGCAACGGTGTGTTT
dorsal (present study)	GGCGTTTGGGGACTTTCAAC
	GGATGGTCGCTTCAGTTGGA
STAT (present study)	TACGGGGACAAATGTGACGG
	CATCTGCAAACAACGCGTCA
attacin (21)	CGACAGGACCCACAAGCATT
	GTCCTTCGGCAGTCAGACTC
defensin 1 (21)	TGTGAGTTGTGACGTCCTGG
	ACTTAACGACGACACCTGCAT
defensin 2 (21)	TTGTTGGAGTCGTCAGTGCT
	CCAAATAGGGCTTCCGGGAA
*Leishmania* actin (30)	GTCGTCGATAAAGCCGAAGGTGGTT
	TTGGGCCAGACTCGTCGTACTCGCT
bacteria 16S rRNA (31)	TCCTACGGGAGGCAGCAGT
	GGACTACCAGGGTATCTAATCCTGTT

*Underlined nucleotides correspond to the T7 promoter.

Gene silencing efficiency was assessed in colony-reared females for three days after dsRNA injection in sucrose-fed insects and for two days after injection in blood-fed insects (**Supplementary Additional File 2**).

### 2.5 RNA extraction and cDNA synthesis

Total RNA extraction from pooled gut samples of *P. perniciosus* females was performed using the High Pure RNA Isolation Kit (Roche), following the manufacturer’s instructions. cDNA synthesis was carried out using the Transcriptor First Strand cDNA Synthesis Kit (Roche) using up to 1 μg of total RNA and a combination of anchored oligo dT_(18)_ and random primers in a 20 μL reaction following the manufacturer’s instructions. The cDNA concentration was quantified using the Quantus fluorometer (Promega).

### 2.6 Gene expression analysis, *Leishmania*, and microbiota quantification by RT-qPCR

The transcriptional profiles of *EcR*, *Eip75B*, *serpent*, *relish*, *dorsal*, *STAT*, *defensin 1*, *defensin 2*, and *attacin* in *P. perniciosus* guts were assessed by quantitative real-time PCR (RT-qPCR) with gene-specific primers (**Table 1**). *P. perniciosus* dorsal, STAT, and Relish sequences were identified and are provided in **Supplementary Additional File 3**. Relative parasite load was estimated between experimental dsEcR and control dsLacZ groups using the *Leishmania* actin gene (30). Relative bacterial abundance was estimated by RT-qPCR using broad-range bacterial 16S rRNA primers described by Nadkarni et al. (31), which amplify a 466-bp fragment corresponding to positions 331–797 of the *Escherichia coli* 16S rRNA gene, on cDNA generated from total RNA extracted from sand fly gut, as a proxy for transcriptionally active bacteria rather than absolute bacterial DNA copy number.

Reactions were carried out with 1 ng of cDNA, gene-specific primers, and SYBR Green Master Mix (Roche) in a final reaction volume of 10 µL on a LightCycler 480 system (Roche). Amplification conditions consisted of an initial activation at 95 °C for 10 min, followed by 45 cycles of denaturation at 95 °C for 10 s, annealing at 60 °C for 20 s, and extension at 72 °C for 45 s. Gene expression levels were normalized against the endogenous reference genes GAPDH (21) and EF-1 (29). Relative quantification was calculated using the Pfaffl method (33), incorporating primer-specific amplification efficiencies, and results were expressed as relative expression values normalized to the corresponding control/calibrator group. The calculated amplification efficiencies are provided in **Supplementary Additional File 4**. The entire experiment was independently repeated three times.

### 2.7 *Leishmania* morphometric analysis

Sand fly guts were dissected at 5 days post *L. infantum* infection. Three smears from dsLacZ-injected control females and three smears from dsEcR-injected females were analyzed, with approximately 30 parasites recorded per smear, resulting in 85 photographed parasites per experimental group. Smears were fixed with methanol, stained with Giemsa, and examined using a Motic inverted microscope equipped with a 100× oil immersion objective and a Moticam PRO S5 Lite camera. Body length, body width, and flagellar length were measured using ImageJ software. Developmental forms were classified according to previously described morphometric criteria (34). Procyclic promastigotes were defined as forms with body length <14 µm and flagellar length shorter than the body length; elongated nectomonads as forms with body length ≥14 µm; metacyclic promastigotes as forms with body length <14 µm and flagellar length longer than twice the body length; and leptomonads as forms with body length <14 µm and flagellar length equal to or longer than the body length but ≤2 times the body length.

### 2.8 Statistical analysis

Data were analyzed using GraphPad Prism software (GraphPad Software, CA, United States). Comparisons of gene expression between experimental and control groups were performed using the nonparametric Mann–Whitney U test, chosen because the datasets were not normally distributed. Comparisons of the number of parasite developmental forms were plotted in a contingency table. Statistical difference was tested using chi-square and Fisher’s exact test. Differences were considered statistically significant at *p* < 0.05.

## 3 Results

### 3.1 EcR silencing impairs the ecdysone signaling pathway in *P. perniciosus* females

**Figure 1:**
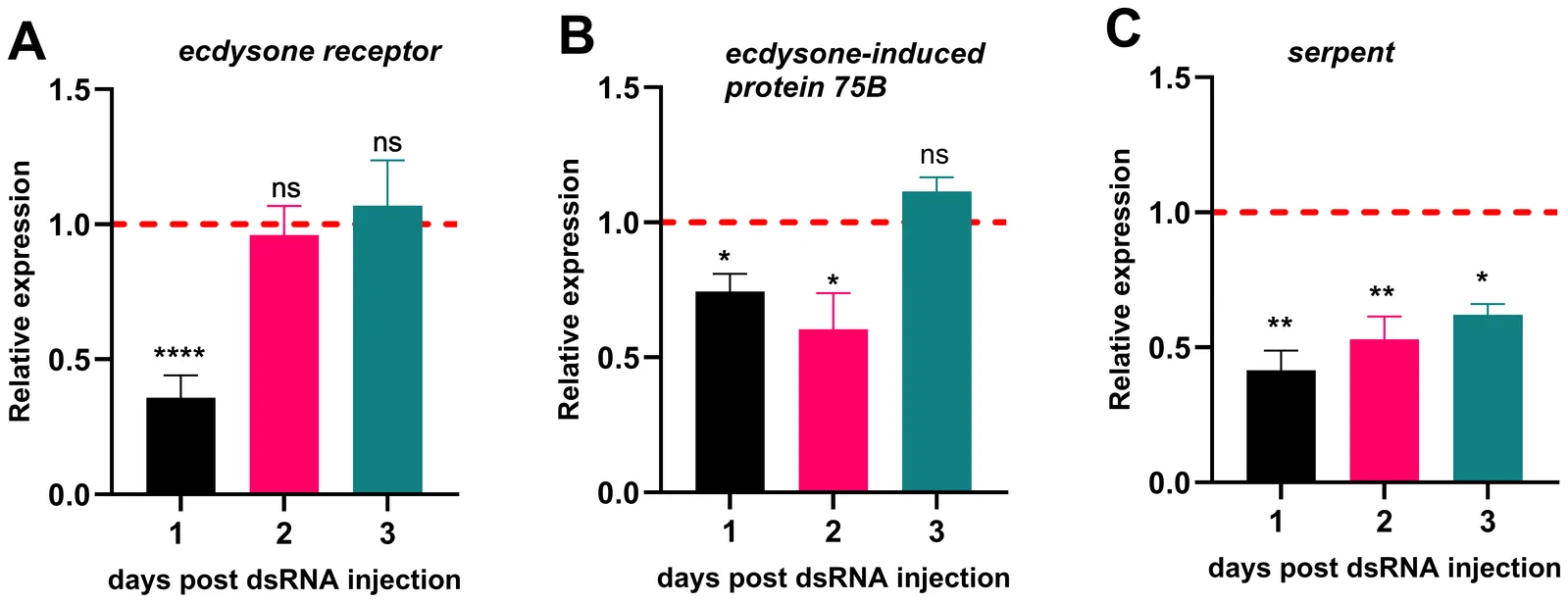
EcR silencing reduces the expression of ecdysone signaling-related genes in *P. perniciosus*. Sand fly females were maintained on a sugar-fed regimen and injected with dsEcR or control dsLacZ. The gene expression of **(A)** *EcR*, **(B)** *Eip75B*, and **(C)** *serpent* was evaluated in guts dissected 1, 2, and 3 dpdi by RT-qPCR. Relative expression values were calculated using the Pfaffl method, incorporating primer-specific amplification efficiencies, and normalized to the dsLacZ-injected control group, which was assigned a value of 1. Bars represent the mean ± SEM of three independent experiments (n = 15). The red dotted line represents the control group injected with dsLacZ, which was assigned a relative expression value of 1 at each time point. Means were compared using the Mann-Whitney test: *p < 0.05, **p < 0.01, and ****p < 0.0001.

To investigate the role of EcR in the hormonal signaling of *P. perniciosus*, we first silenced EcR in recently emerged females. The effects of EcR knockdown were assessed by quantifying the expression of EcR and downstream ecdysone-responsive genes *Eip75B* and *serpent*. *EcR* expression was significantly reduced in the guts of dsEcR-injected females compared to dsLacZ-injected females at 1 day post dsRNA injection (dpdi) (**Figure 1A**), confirming effective EcR silencing; however, transcript levels recovered to control levels at 2 and 3 dpdi. Statistically significant changes in *Eip75B* expression were observed only at 1 and 2 dpdi (**Figure 1B**). *Serpent* expression was significantly downregulated at all evaluated time points following *EcR* silencing (**Figure 1C**).

### 3.2 EcR silencing impairs the Imd and JAK/STAT pathways, affecting AMPs expression and microbiota population

**Figure 2:**
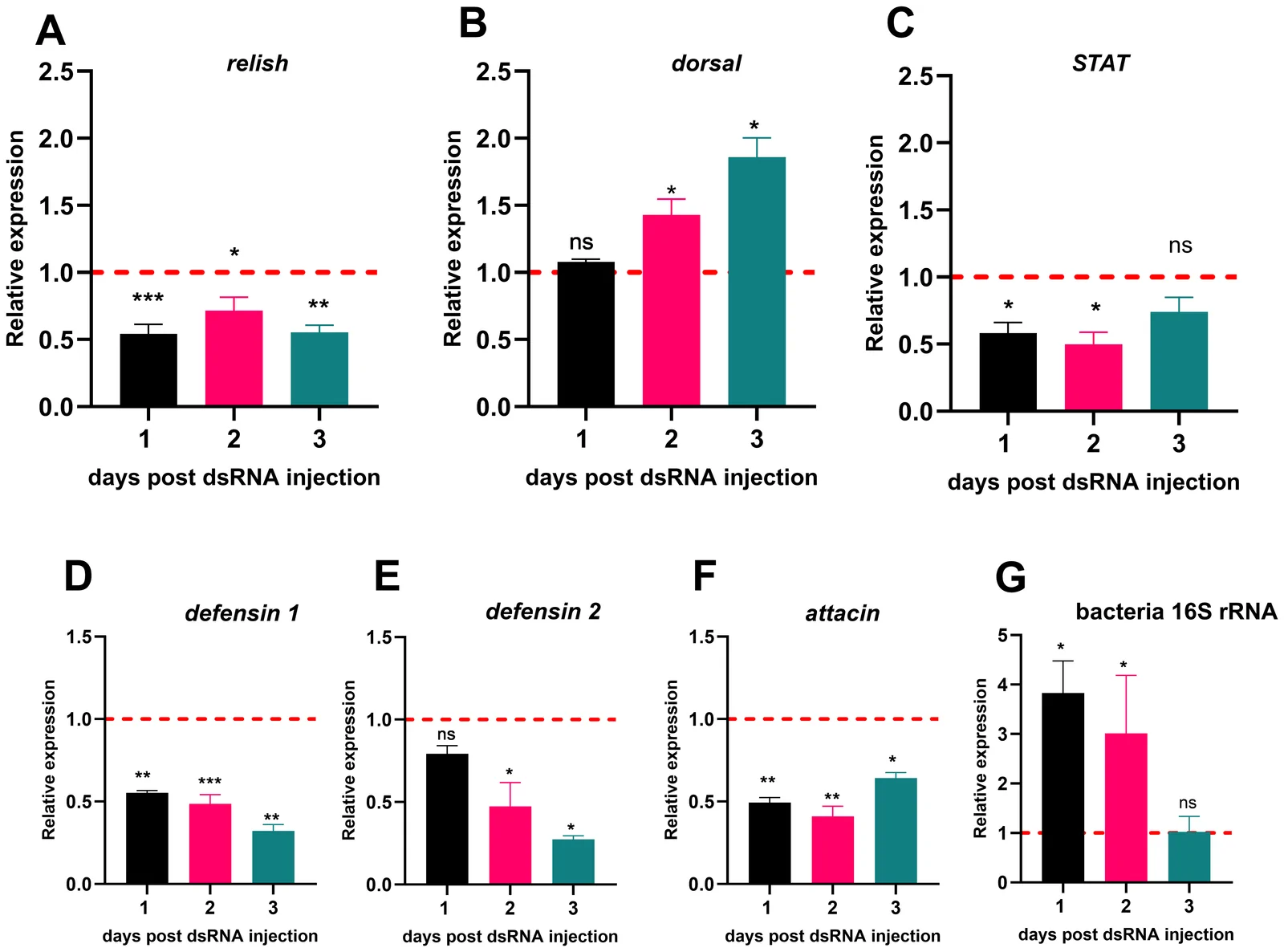
EcR silencing alters the expression of immune-related genes. *P. perniciosus* females were maintained under a sugar-fed regimen. The gene expression of **(A)** *relish*, **(B)** *dorsal*, **(C)** *STAT*, **(D)** *defensin 1*, **(E)** *defensin 2*, **(F)** *attacin*, and **(G)** bacteria 16S rRNA was evaluated in dissected guts 1, 2, and 3 dpdi by RT-qPCR. Relative expression values were calculated using the Pfaffl method, incorporating primer-specific amplification efficiencies, and normalized to the dsLacZ-injected control group, which was assigned a value of 1. Bars represent the mean ± SEM of three independent experiments (n = 15). The red dotted line represents the time-matched dsLacZ-injected control group, which was assigned a relative expression value of 1 at each time point. Asterisks above bars indicate statistically significant differences between dsEcR-injected females and their corresponding time-matched dsLacZ-injected controls. Means were compared using the Mann–Whitney test: *p < 0.05, **p < 0.01, and ***p < 0.001.

To investigate the potential effects of EcR silencing in other immunity-related genes in sugar-fed females, we selected three pathway-related transcription factors and AMPs and assessed their gene expression in *P. perniciosus* gut. *Relish* expression was significantly reduced at all evaluated time points when compared to dsLacZ-injected controls (**Figure 2A**). *Dorsal* expression remained like control at 1 dpdi but was significantly upregulated at both 2 and 3 dpdi (**Figure 2B**). *STAT* transcripts were downregulated at 1 dpdi and 2 dpdi, while no difference was observed at 3 dpdi (**Figure 2C**). Among AMPs, *defensin 1* was consistently suppressed throughout the experiment (**Figure 2D**). *Defensin 2* showed no change at 1 dpdi but was significantly reduced at 2 and 3 dpdi (**Figure 2E**). *Attacin* expression was downregulated at all analyzed time points (**Figure 2F**). In addition, we assessed the potential effect on bacteria load in the sand fly gut. The expression of bacteria 16S rRNA was increased on 1 and 2 dpdi (**Figure 2G**).

### 3.3 *Leishmania* infection upregulates ecdysone signaling-related genes in *P. perniciosus*

To evaluate whether blood feeding modulates ecdysone signaling, we analyzed the expression of ecdysone-related genes in the guts of *P. perniciosus* females on different days post blood meal. *EcR* expression increased significantly from 1 to 3 days post blood feeding (DPBF) compared with non-fed control guts (**Supplementary Additional File 1A**). The downstream target *Eip75B* exhibited a more pronounced response, with significant upregulation from 1 to 5 DPBF (**Supplementary Additional File 1B**), indicating that blood feeding alone is sufficient to activate ecdysone signaling in the sand fly gut. Because *EcR* is upregulated after blood feeding, we tested whether prior blood feeding could affect RNAi efficiency. The dsEcR injection at 24 hours after a blood meal resulted in a significant reduction in *EcR* expression on the two following days, while *Eip75B* and *serpent* were downregulated on the next day (**Supplementary Additional File 2**), indicating that RNAi-mediated suppression was preserved under blood-fed conditions.

**Figure 3:**
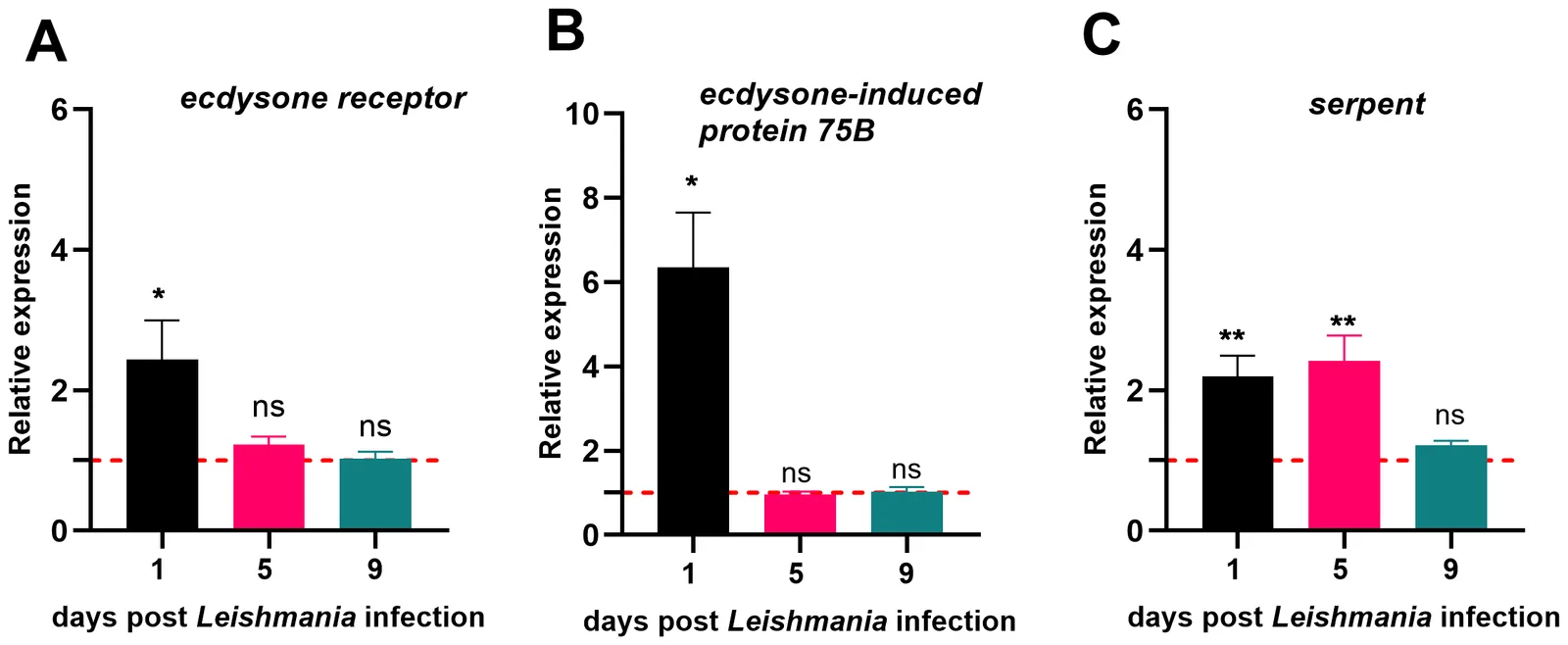
Effects of *L. infantum* ingestion on the expression of ecdysone-signaling-related genes in *P. perniciosus*. Sand fly females were fed on blood seeded with *L. infantum*. The gene expression of **(A)** *EcR*, **(B)** *Eip75B*, and **(C)** *serpent* was evaluated in dissected guts 1, 5, and 9 DPLI by RT-qPCR. Results are expressed as the mean ± SEM of three independent experiments (n = 15). The red dotted line represents the control/calibrator group, fed on blood only, which was assigned a relative expression value of 1 at each time point. Relative expression values were calculated using the Pfaffl method, incorporating primer-specific amplification efficiencies. Statistical differences were analyzed using the Mann-Whitney test: *p < 0.05 and **p < 0.01.

In parallel, we investigated whether *Leishmania* infection could affect genes related to ecdysone signaling in *P. perniciosus* guts. *EcR* and *Eip75B* were significantly upregulated at 1 day post *Leishmania* infection (DPLI) compared to blood-fed controls (**Figures 3A, B**), but their expression returned to control levels at 5 and 9 DPLI. In addition, *serpent* expression was significantly increased at both 1 and 5 DPLI (**Figure 3C**), then returning to control levels by 9 DPLI.

### 3.4 *Leishmania infantum* infection triggers Imd, Toll, and JAK/STAT pathways and alters microbiota in the sand fly gut

**Figure 4:**
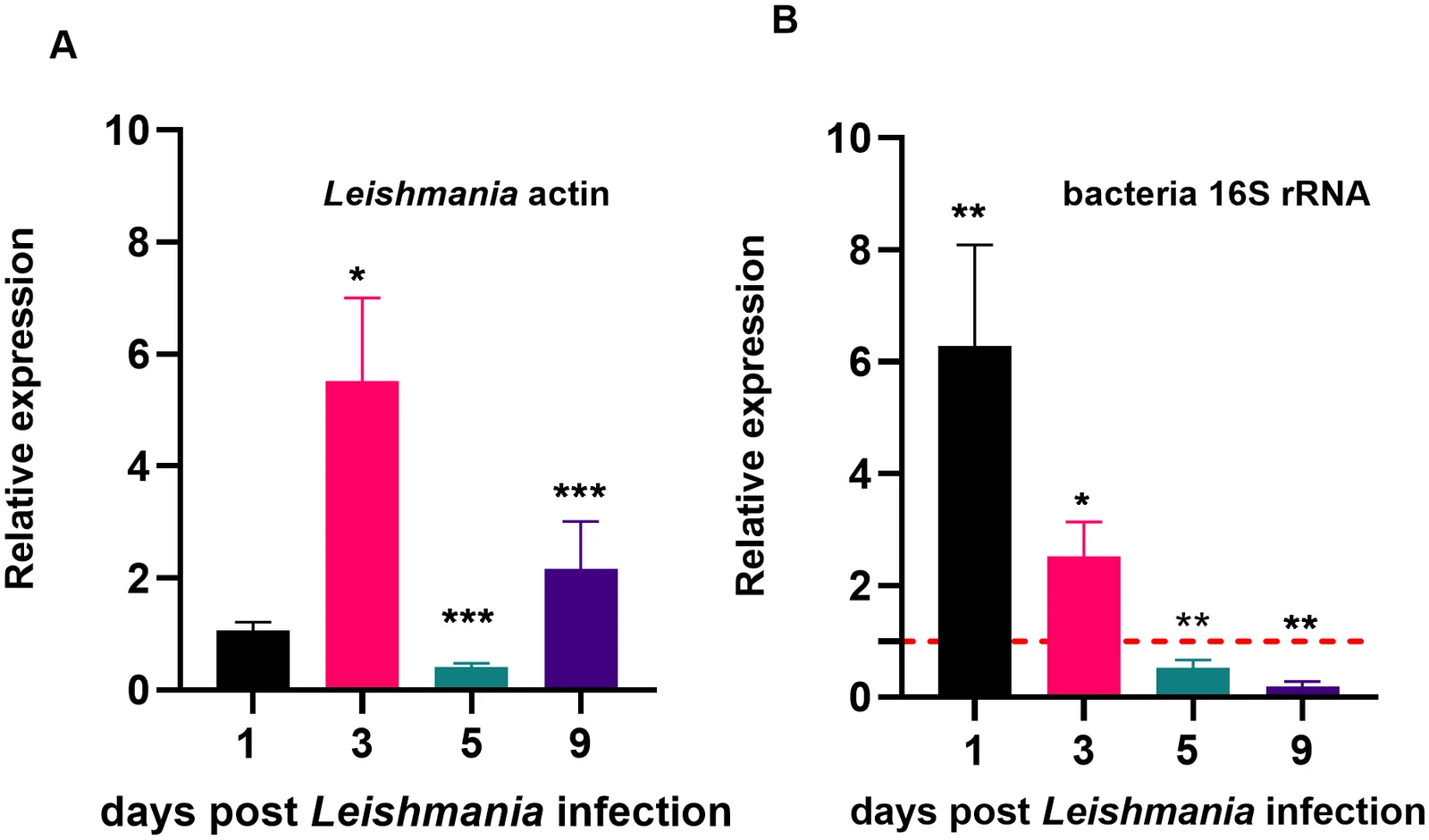
Effects of *Leishmania* infection on *total gut microbiota*. Sand fly females were fed on blood seeded with *L. infantum*. The gene expression of A) *Leishmania actin*, B) bacteria 16S rRNA was evaluated in dissected guts at 1, 3, 5, and 9 DPLI by RT-qPCR. In panel A, *Leishmania* actin values were normalized to the infected 1 DPLI group, which was used as the calibrator to assess parasite abundance over time. In panel B, bacteria 16S rRNA values were normalized to the corresponding time-matched blood-fed uninfected control group, which was assigned a relative expression value of 1 and is represented by the red dotted line. Results are expressed as the mean ± SEM of three independent experiments (n = 15). Relative expression values were calculated using the Pfaffl method, incorporating primer-specific amplification efficiencies. Statistical differences were analyzed using the Mann–Whitney test: *p < 0.05, **p < 0.01, and ***p < 0.001.

We also investigated whether *Leishmania* infection alters the expression of immunity-related genes in *P. perniciosus* guts. Intensity of *L. infantum* infection was assessed by measuring *Leishmania* actin transcripts. Compared with 1 day post *Leishmania* infection (DPLI), parasite abundance increased approximately six-fold at 3 DPLI, followed by a significant decline at 5 DPLI. By 9 DPLI, parasite levels rose again, reaching approximately two-fold higher than at 1 DPLI and five-fold higher than at 5 DPLI (**Figure 4A**).

In parallel, we studied the effects of *L. infantum* infection on the total bacterial population in the gut of *P. perniciosus*. At 1 DPLI, a significant increase in total bacterial load was observed in *Leishmania*-infected females, reaching nearly 6-fold higher levels compared to uninfected females (**Figure 4B**). By 3 DPLI, the bacterial population was still higher than in uninfected females (**Figure 4B**), but a clear decrease was observed in comparison to infected females at 1 DPLI. At 5 DPLI, a significant decline in bacterial 16S rRNA levels was detected, which persisted until 9 DPLI (**Figure 4B**).

**Figure 5:**
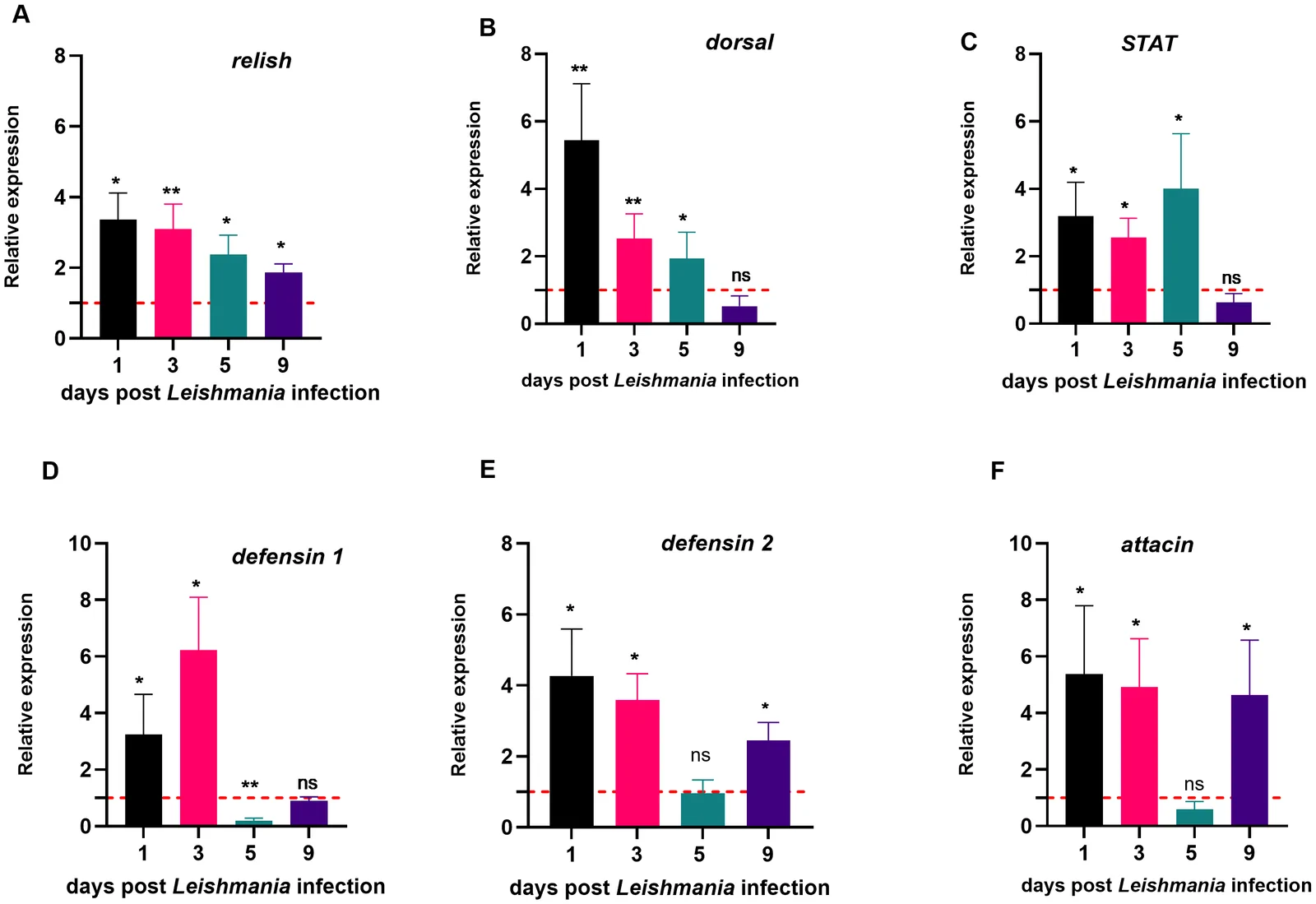
Effects of Leishmania infection on P. perniciosus immune-related genes. Sand fly females were fed on blood seeded with *L. infantum*. The gene expression of A) *relish* B) *dorsal*, C) *STAT*, D) *defensin 1*, E) *defensin 2*, F) *attacin* was evaluated in dissected guts 1, 3, 5, and 9 DPLI by RT-qPCR. Results are expressed as the mean ± SEM of three independent experiments (n = 15). The red dotted line represents the control/calibrator group fed on blood only, which was assigned a relative expression value of 1 at each time point. Relative expression values were calculated using the Pfaffl method, incorporating primer-specific amplification efficiencies. Statistical differences were analyzed using the Mann-Whitney test: *p < 0.05 and **p < 0.01.

The expression of *P. perniciosus* immune-related genes was modulated following *L. infantum* infection. The gene expression of *relish, dorsal*, and *STAT* was significantly upregulated after *L. infantum* infection at 1, 3, and 5 DPLI compared to blood-fed control females (**Figures 5A–C**). At 9 DPLI, *relish* transcripts remain higher when compared to uninfected blood-fed females (**Figure 5A**). However, *dorsal* and *STAT* gene expression decrease, reaching similar levels to uninfected guts (**Figures 5B, C**).

In addition, we studied the effects of *L. infantum* infection on the sand fly immune effectors. The AMP genes *defensin 1*, *defensin 2*, and *attacin* were significantly upregulated at 1 and 3 DPLI (**Figures 5D, E, F**). By 5 DPLI, a significant reduction in *defensin 1* expression was observed compared to blood-fed controls (**Figure 4F**). *Defensin 2* and *attacin* levels showed a tendency to diminish at 5 DPLI when compared to 1 and 3 DPLI (**Figures 5E, F**). At 9 DPLI, *defensin 2* and *attacin* expression increased again compared to non-infected guts (**Figures 4G, H**).

### 3.5 EcR silencing affects the activation of ecdysone signaling after *Leishmania* infection in *P. perniciosus*

**Figure 6:**
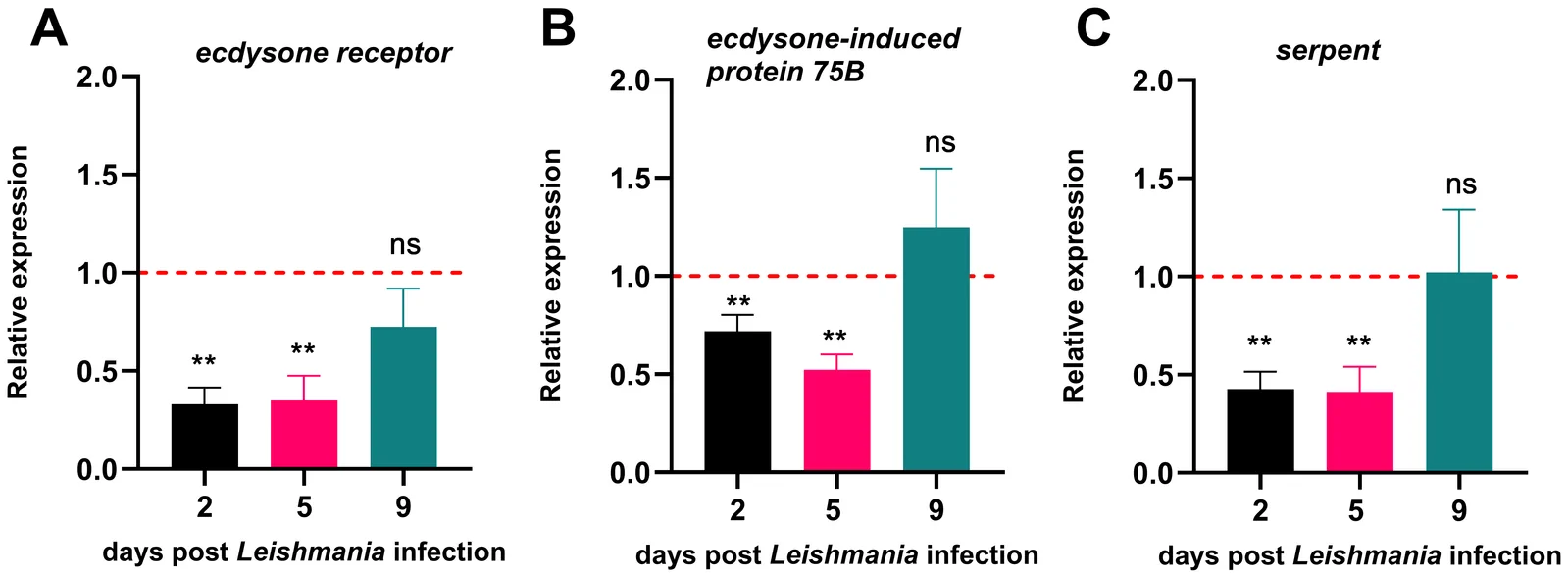
Impact of EcR silencing and its effects on downstream ecdysone-signaling genes following *L. infantum* infection. All *P. perniciosus* females were infected with *Leishmania* and injected with dsEcR or dsLacZ 24 hours post-infection. The expression levels of **(A)** *EcR*, **(B)** *Eip75B*, and **(C)** *serpent* were assessed in dissected guts 2, 5, and 9 DPLI by RT-qPCR. Data is presented as the mean ± standard error of the mean (SEM) from three independent experiments (n = 15 per group). The red dotted line represents the control group injected with dsLacZ, which was assigned a relative expression value of 1 at each time point. Relative expression values were calculated using the Pfaffl method, incorporating primer-specific amplification efficiencies. Statistical differences between dsEcR and control groups were determined using the Mann-Whitney test: **p < 0.01.

Given that *L. infantum* infection upregulated ecdysone- and immunity-related genes in *P. perniciosus*, we next investigated whether suppressing EcR expression in infected sand flies would affect expression of downstream ecdysone-signaling genes. *EcR* was significantly downregulated at 2 and 5 DPLI compared to the control group injected with dsLacZ, confirming gene silencing; nevertheless, its expression returned to control levels at 9 DPLI (**Figure 6A**). A similar transient induction was observed for *Eip75B* and *serpent*, which decreased significantly at 2 and 5 DPLI, then returned to control levels by 9 DPLI (**Figures 6B, C**).

### 3.6 Ecdysone signaling disruption alters the expression of immune-related genes and parasite-microbiota dynamics

**Figure 7:**
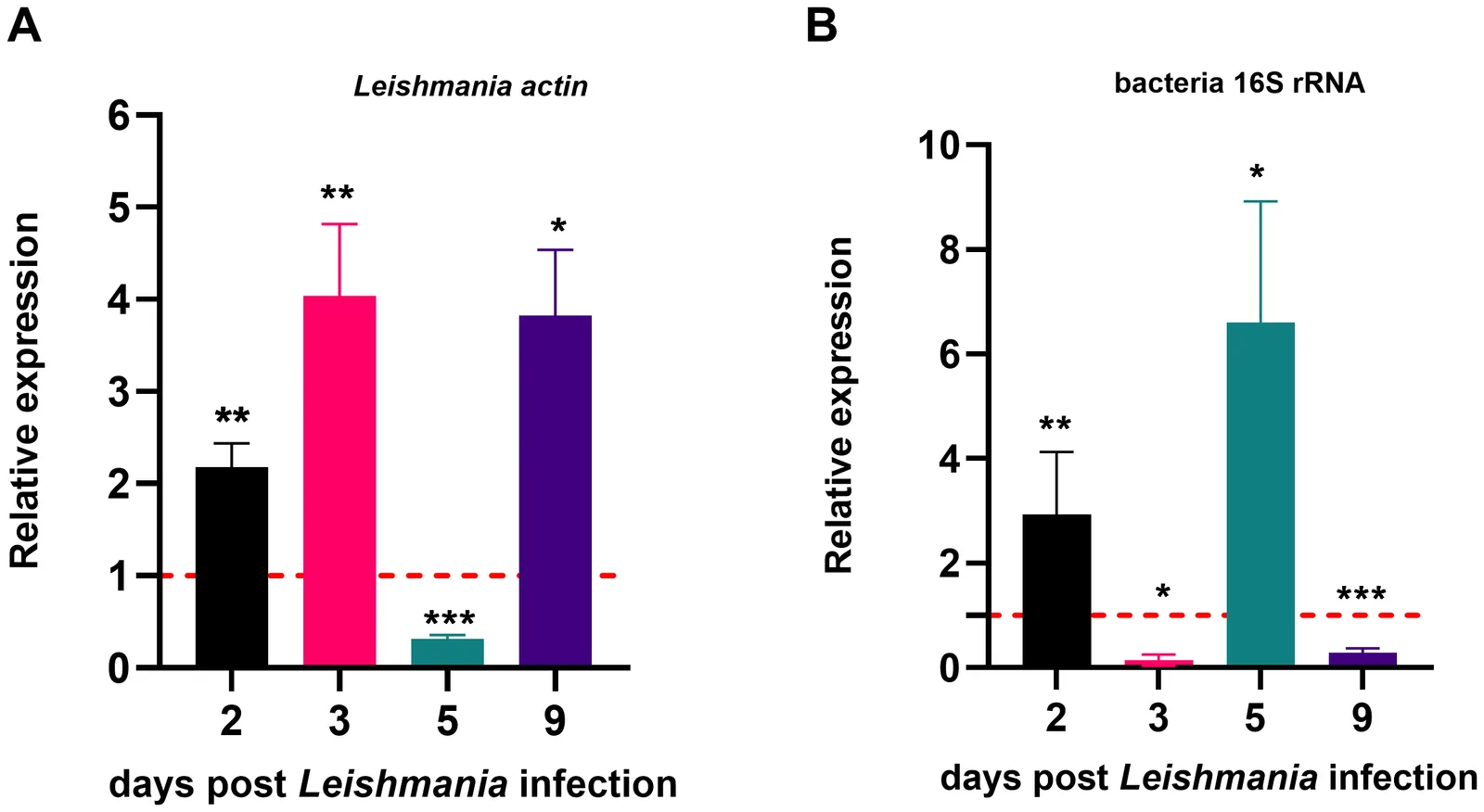
EcR silencing alters Leishmania infection and total gut microbiota. *P. perniciosus* females were infected with *L. infantum* and injected with dsEcR or dsLacZ 24 hours post-infection. The expression levels of A) *Leishmania actin*, B) bacteria 16S rRNA were evaluated in dissected guts at 2, 3, 5, and 9 DPLI by RT-qPCR. Results are expressed as the mean ± SEM of three independent experiments (n = 15). The red dotted line represents the control group injected with dsLacZ, which was assigned a relative expression value of 1 at each time point. Relative expression values were calculated using the Pfaffl method, incorporating primer-specific amplification efficiencies. Statistical differences between dsEcR and control groups were determined using the Mann-Whitney test: *p < 0.05, **p < 0.01, and ***p < 0.001.

Finally, we investigated the effect of EcR silencing on parasite abundance, expression of immunity-related genes, and gut bacteria in infected sand flies. Parasite abundance in infected dsEcR sand flies was two- and fourfold higher at 2 and 3 DPLI, respectively, compared to the control group. In contrast, parasite load was significantly reduced at 5 DPLI, followed by a fourfold increase at 9 DPLI in the infected dsEcR group (**Figure 7A**). The effect on bacterial load in infected dsEcR insects varied over time, with an increase at 2 and 5 DPLI and a decrease at 3 and 9 DPLI (**Figure 7B**).

**Figure 8:**
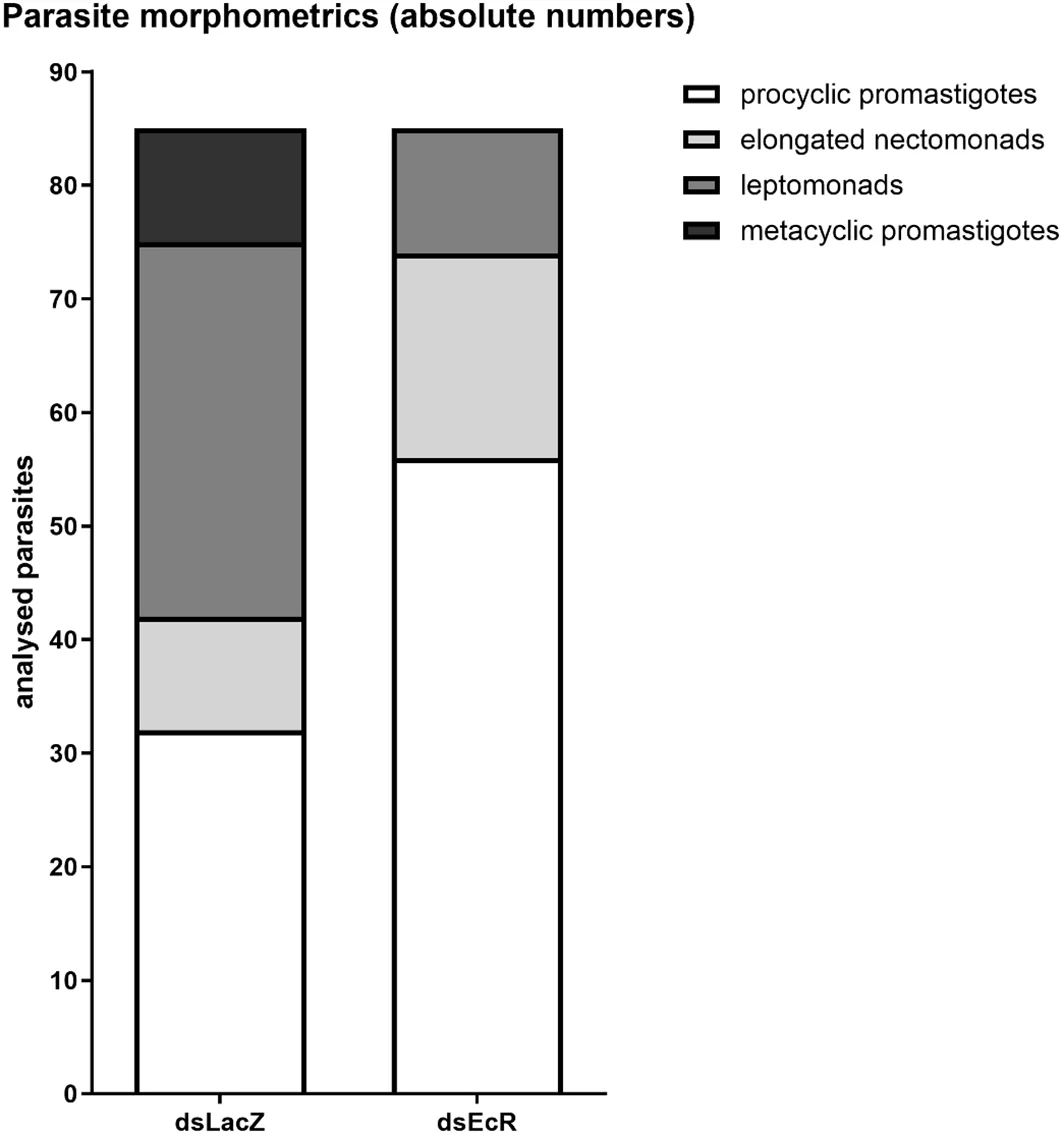
EcR silencing alters *Leishmania infantum* development in *Phlebotomus perniciosus* gut. Sand fly guts were dissected at 5 days post *L. infantum* infection, reflecting a mid-stage infection. Three gut smears from either dsLacZ- or dsEcR-injected females were Giemsa-stained, and approximately 30 parasites per smear were photographed. In total, 85 images per group were analyzed. The number of parasite developmental forms was plotted in a contingency table. Statistical difference was tested using chi-square and Fisher’s exact test (p < 0.0001).

Morphological analyses performed at 5 DPLI revealed altered parasite developmental distribution in dsEcR insects, characterized by an increased proportion of procyclic promastigotes together with reduced leptomonad and no detection of metacyclic forms (**Figure 8**).

**Figure 9:**
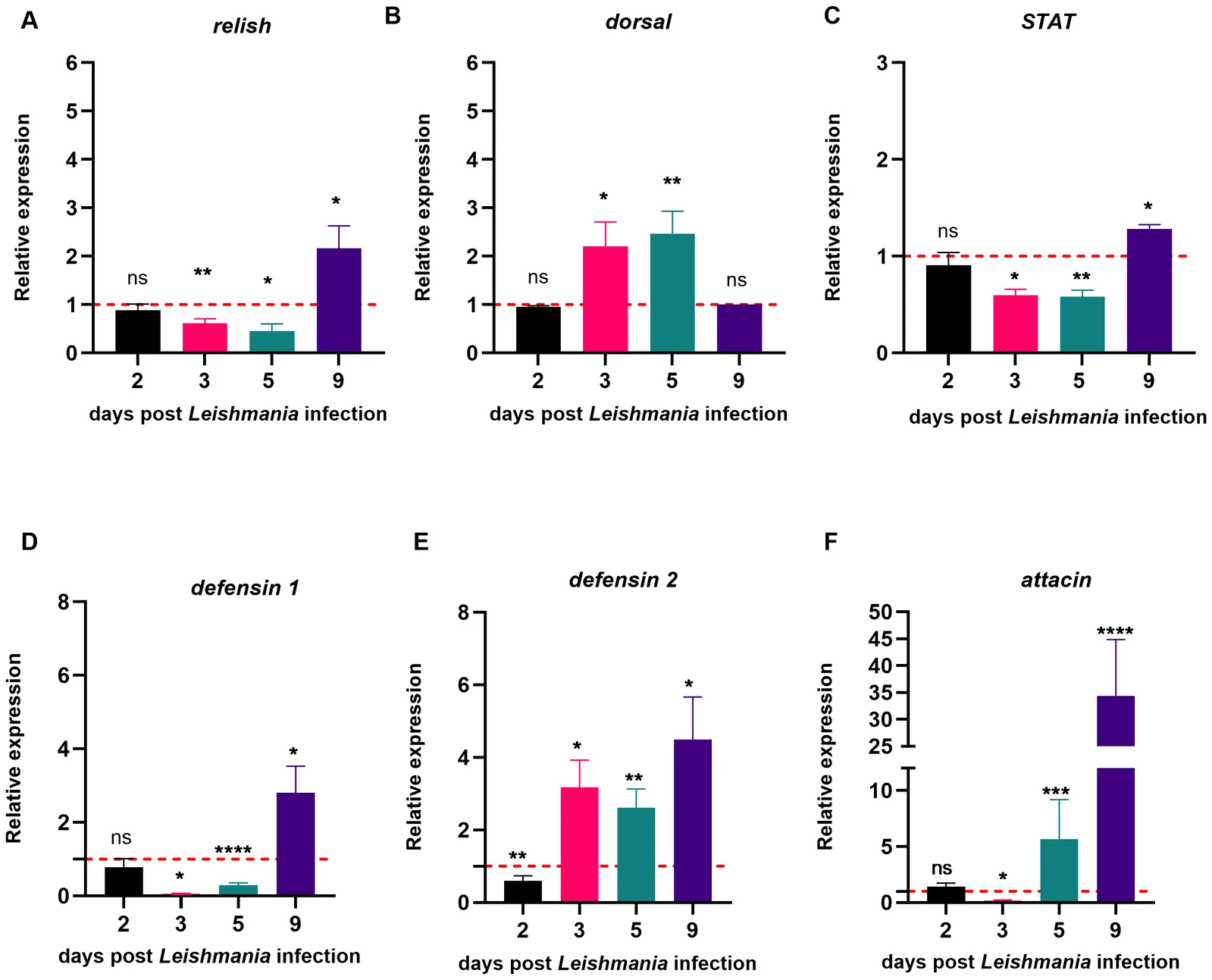
EcR silencing alters the expression of *P. perniciosus* immune-related genes during *Leishmania infantum* infection. *P. perniciosus* females were infected with *L. infantum* and injected with dsEcR or dsLacZ 24 hours post-infection. The expression levels of A) *relish* B) *dorsal*, C) *STAT*, D) *defensin 1*, E) *defensin 2*, F) *attacin* were evaluated in dissected guts at 2, 3, 5, and 9 days after *Leishmania* infection by RT-qPCR. Results are expressed as the mean ± SEM of three independent experiments (n = 15). The red dotted line represents the control group injected with dsLacZ, which was assigned a relative expression value of 1 at each time point. Relative expression values were calculated using the Pfaffl method, incorporating primer-specific amplification efficiencies. Statistical differences between dsEcR and control groups were determined using the Mann-Whitney test: *p < 0.05, **p < 0.01, and ****p < 0.0001.

Both *relish* and *STAT* expression were downregulated while *dorsal* was upregulated at 3 and 5 DPLI (**Figures 9A, B, C**). Regarding AMPs expression in EcR-silenced infected females, *defensin 1* was downregulated at 3 and 5 DPLI, while upregulated at 9 DPLI (**Figure 9D**). The *defensin 2* expression decreased at 2 DPLI, but its gene abundance was significantly upregulated at 3, 5, and 9 DPLI (**Figure 9E**). *Attacin* expression was reduced at 3 DPLI and increased at 5 and 9 DPLI (**Figure 9F**).

## 4 Discussion

The establishment of *Leishmania* infection in the sand fly gut depends on the regulation of immune responses and microbial homeostasis (22, 23). Our previous work demonstrated that disruption of ecdysone signaling suppresses antimicrobial peptide (AMP) expression in *P. perniciosus*, suggesting a link between hormonal signaling and immune regulation in sand flies (21). Here, we further investigated the role of EcR-mediated signaling in the regulation of gut immunity, microbiota dynamics, and *L. infantum* development.

Transcriptomic studies in *P. perniciosus* have identified conserved innate immune and endocrine signaling pathways (35, 36). In other insect vectors, ecdysone signaling modulates immune responses and pathogen susceptibility (14–16, 18). Our results support a similar role in sand flies and indicate that EcR acts as an important upstream regulator integrating endocrine and immune signaling.

EcR silencing in sugar-fed females effectively disrupted the hormonal signaling cascade, as demonstrated by reduced *EcR* transcript levels together with suppression of the ecdysone-responsive genes *Eip75B* and *serpent*. In holometabolous insects, *Eip75B* is a classical early-response gene induced by 20-hydroxyecdysone during tissue remodeling and serves as a marker of canonical EcR/USP-mediated transcriptional activation (37, 38). In contrast, *serpent*, a GATA transcription factor associated with immune competence and hemocyte differentiation in *D. melanogaster* (39), remained persistently downregulated after EcR silencing. This finding suggests that EcR signaling contributes to maintaining basal immune readiness in the sand fly gut, even in the absence of blood feeding. Similar maintenance of ecdysone-dependent gene expression under non-feeding conditions has also been reported in *Drosophila* and *Anopheles* (5, 14). Disruption of EcR signaling in sugar-fed females also affected key immune pathways involved in gut homeostasis. EcR knockdown reduced the expression of *relish* and *STAT*, indicating impairment of the imd and JAK/STAT pathways, whereas upregulation of *dorsal* was insufficient to restore AMP expression. These findings reinforce the importance of the imd pathway as a major regulator of intestinal AMPs in sand flies, consistent with observations in *D. melanogaster* (40–42). Moreover, AMP promoters in *D. melanogaster* are frequently regulated through cooperation between κB transcription factors and GATA-binding proteins such as Serpent (41, 43), suggesting that persistent suppression of *serpent* may further compromise immune responsiveness in *P. perniciosus*. Importantly, these immune alterations were accompanied by increased bacterial 16S rRNA relative abundance in sugar-fed EcR-silenced females, indicating that EcR signaling contributes to maintain basal gut immune homeostasis independently of blood feeding or parasite infection. This interpretation is consistent with studies in *Drosophila* showing that Imd-dependent AMP production helps regulate gut bacterial populations and maintain intestinal homeostasis (40, 44). Thus, the reduction of *relish*, *STAT*, and AMP transcripts, together with increased bacterial 16S rRNA abundance, supports a model in which EcR acts upstream of gut immune signaling, particularly the Imd pathway and, at least in part, JAK/STAT signaling, thereby modulating immune effector expression and contributing to microbiota homeostasis in *P. perniciosus*.

Following *L. infantum* infection, genes associated with ecdysone signaling were upregulated during the early phase of infection, highlighting the importance of activating the hormonal pathway in response to parasite establishment. Because 20E levels were not directly quantified in this study, ecdysone signaling activation was inferred from transcriptional changes in EcR and downstream ecdysone-responsive genes, including Eip75B and serpent. Previous work in *L. longipalpis* demonstrated that *L. infantum* infection modulates genes associated with hormone metabolism, including ecdysone-related pathways, particularly during blood digestion and early parasite development (45). Similar interactions between parasites and endocrine signaling have been reported in other vector systems. In mosquitoes, *Plasmodium* infection alters ecdysone signaling and downstream immune responses (15, 46). In triatomines, the neuroendocrine system and ecdysone signaling regulate gut physiology, immune homeostasis (17), and *Trypanosoma cruzi* development within the vector (47). These observations support the hypothesis that parasites exploit hormonally regulated physiological states of the vector to establish permissive environments for survival and transmission.

Our results further showed that *Leishmania* infection activated transcription factors from the IMD, Toll, and JAK/STAT pathways, accompanied by increased AMP expression during the initial stages of infection. However, comparison with previous studies suggests that these responses are species- and tissue-dependent. In *P. papatasi*, *dorsal* but not *relish* was induced following *L. major* infection under bacteria-depleted conditions (48), whereas whole-body analyses of *L. longipalpis* infected with *L. infantum* showed no significant changes in *STAT* expression (49). These differences highlight the importance of gut-specific immune regulation in sand flies.

The AMP expression profiles observed here also indicate a dynamic interaction between parasite infection and gut microbiota. *Defensin 1* expression closely paralleled parasite detection, whereas *defensin 2* and *attacin* were associated with fluctuations in both bacterial and parasite abundance. Similar variability in AMP regulation has been reported in different sand fly–*Leishmania* systems (48, 50–52), suggesting that AMP responses are shaped by both parasite-derived and bacterial stimuli.

*Leishmania* parasites are also known to manipulate vector immune responses to facilitate their establishment and development. In *L. longipalpis*, *L. infantum* suppresses the JAK/STAT pathway in LL5 cells (49), and the parasite metalloprotease GP63 induces the expression of SHP (53), a negative regulator of Toll and JAK/STAT signaling (54). Silencing SHP reduces parasite loads during early infection, demonstrating that parasite-induced immune repression promotes infection establishment (53). In this context, the temporal activation of *relish*, *STAT*, and AMP genes observed in our study likely reflects a finely tuned balance between vector immune activation and parasite-mediated immune modulation. Our findings further suggest that EcR signaling functions upstream of these processes, coordinating endocrine and immune responses during infection.

Functional disruption of EcR signaling during *Leishmania* infection altered both immune regulation and parasite development. Because EcR transcript levels recovered at later time points, particularly by 9 DPLI, the effects observed during infection should be interpreted mainly within the early-to-mid window of EcR disruption, when EcR, Eip75B, and serpent expression were still reduced. EcR silencing reduced the expression of *relish* and *STAT*, supporting the view that IMD and JAK/STAT signaling are, at least in part, regulated downstream of ecdysone signaling. During the early phase of infection, reduced expression of *defensin 1* and *attacin* coincided with increased parasite abundance and decreased bacterial abundance, suggesting that transient immune suppression initially favors parasite establishment, while simultaneously affecting bacterial populations. Similar observations were reported in *P. papatasi*, where suppression of *defensin 1* increased the abundance of proliferative forms of *L. major* (24).

During the mid-stage of infection in *P. perniciosus*, canonical Imd signaling remained impaired; however, some AMPs, particularly defensin 2 and attacin, became upregulated. This pattern suggests the activation of compensatory or non-canonical immune mechanisms, similar to those described in *D. melanogaster* (12). At the same time, reduced parasite loads in EcR-silenced sand flies coincided with increased bacterial abundance, suggesting that altered AMP regulation disrupts the balance between parasite persistence and microbiota homeostasis. Morphometric analyses of parasites (34) in mid-stage infection of *P. perniciosus*, when gene silencing is still efficient, further revealed a delayed *Leishmania* development in EcR-silenced sand flies. Under these conditions, it is difficult to identify the main drivers between AMP regulation and parasite-microbiota balance in the sand fly gut. Nevertheless, because gut microbial homeostasis is essential for proper *Leishmania* differentiation, and both increases or decreases in bacterial abundance influence parasite growth and metacyclogenesis (55, 56), our results suggest that microbiota dysregulation contributes to the observed effects on both parasite abundance and development in *P. perniciosus*. During infection, altered immune gene expression and AMP profiles were associated with shifts in the gut microbiota and *Leishmania* dynamics, including opposing temporal trends at specific stages. Although this association should not be interpreted as strictly proportional or directly causal, it supports the hypothesis that EcR-dependent immune regulation helps shape the gut environment, thereby affecting both microbial balance and parasite development. Because the present study focused on EcR-dependent changes in relative bacterial 16S rRNA abundance, future microbiome-resolved analyses will help determine which bacterial taxa are affected by EcR disruption and how specific microbial groups contribute to *Leishmania* development in the sand fly gut.

Overall, our data support a model in which *L. infantum* benefits from a controlled activation of ecdysone signaling that fine-tunes gut immune responses and prevents excessive bacterial proliferation. When EcR signaling is disrupted, this regulatory balance is lost, leading to microbiota overgrowth that may exacerbate competition for gut resources and niche occupancy within the gut environment. Comparable interactions between parasite development and ecdysone signaling have been reported in *Anopheles*–*Plasmodium* systems, where altered EcR activity affects parasite growth and development (15, 57). However, while impaired ecdysone signaling in mosquitoes primarily favors parasite growth, our results indicate that in sand flies the outcome is more complex and strongly influenced by interactions with the gut microbiota.

These findings highlight the ecdysone signaling cascade as a potential target for future vector-control strategies, including approaches based on insect growth regulators (IGRs). In sand flies, azadirachtin-mediated disruption of ecdysone signaling alters immune gene expression (21), and our results further show that EcR disruption impairs *Leishmania* development, including differentiation toward transmissible forms. Given the pleiotropic role of ecdysone signaling in insect development, reproduction, immunity, and physiology (1, 2, 18), such approaches would require careful evaluation of stage specificity, vector specificity, delivery strategies, and potential effects on non-target arthropods.

Taken together, our findings demonstrate that ecdysone signaling plays a central role in coordinating immune responses and microbial homeostasis in *P. perniciosus*. We propose that *L. infantum* exploits this hormonal–immune axis to establish a permissive gut environment by modulating imd- and JAK/STAT-associated responses while influencing bacterial competition. Disruption of this signaling network results in microbial dysregulation and ultimately compromises parasite development. These findings provide new insights into the integration of endocrine and immune signaling in sand flies and highlight hormonal pathways as potential targets for strategies aimed at disrupting parasite transmission.

## 5 Conclusion

Collectively, these results identify ecdysone signaling as an upstream regulator of immune and microbial homeostasis during *Leishmania* infection in *P. perniciosus*. By linking endocrine signaling to gut immunity and parasite development, this study highlights the importance of hormonal regulation in shaping sand fly vector competence and reveals EcR signaling as a potential target for strategies aimed at interfering with parasite establishment in insect vectors.

## Data Availability

The datasets presented in this study can be found in online repositories. The names of the repository/repositories and accession number(s) can be found in the article/**Supplementary Material**.
