# Supplementary material for: Ecdysone signaling acts as a regulator of immunity and microbiota balance, shaping Leishmania infantum infection in Phlebotomus perniciosus
Source: Front Immunol. 2026 Aug 11;17:1904500. doi: 10.3389/fimmu.2026.1904500 (PMC13503605; doi:10.3389/fimmu.2026.1904500)
Supplement: Supplementary file 1 [file Table1.docx]

**Additional file 1: Impact of blood ingestion on downstream ecdysone-signaling gene.** *P. perniciosus* females were fed on blood, and non-fed females were used as a control group. The gene expression of (A) *EcR*, (B) *Eip75B*, and (C) *serpent* was evaluated in guts dissected at 1, 2, 3, and 5 days post blood feeding by RT-qPCR and expressed as fold change compared to the non-fed control group. Results are expressed as the mean ± SEM of three independent experiments (n = 15). Statistical differences were analyzed using the Mann-Whitney test: *p < 0.05, **p < 0.01.
