# Supplementary material for: Ecdysone signaling acts as a regulator of immunity and microbiota balance, shaping Leishmania infantum infection in Phlebotomus perniciosus
Source: Front Immunol. 2026 Aug 11;17:1904500. doi: 10.3389/fimmu.2026.1904500 (PMC13503605; doi:10.3389/fimmu.2026.1904500)
Supplement: Supplementary file 2 [file Table2.docx]

 **Additional file 2: Impact of EcR silencing and its effects on downstream ecdysone-signaling genes following blood meal.** All *P. perniciosus* females were fed on blood, then injected with dsEcR (experimental group) or dsLacZ (control group) 24 hours post blood meal. The gene expression of (A) *EcR*, (B) *Eip75B*, and (C) *serpent* was evaluated in dissected guts 1 and 2 days post dsRNA injection by RT-qPCR and expressed as fold change compared to the dsLacZ control group. Results are expressed as the mean ± SEM of three independent experiments (n = 15). The red dotted line represents the control group fed on blood only. Statistical differences were analyzed using the Mann-Whitney test: *p < 0.05, **p < 0.01.
