# Supplementary material for: Ecdysone signaling acts as a regulator of immunity and microbiota balance, shaping Leishmania infantum infection in Phlebotomus perniciosus
Source: Front Immunol. 2026 Aug 11;17:1904500. doi: 10.3389/fimmu.2026.1904500 (PMC13503605; doi:10.3389/fimmu.2026.1904500)
Supplement: Supplementary file 3 [file Table3.docx]

*P. perniciosus* sequences

Relish

>c26379_g2_i4 len=3005 path=[4906:0-240 4192:241-341 5247:342-343 5249:344-418 5321:419-446 267:447-691 10360:692-713 5613:714-799 5698:800-818 580:819-1001 5899:1002-1016 5914:1017-1022 5920:1023-1102 25:1103-1127 50:1128-1214 6106:1215-1258 6149:1259-1260 6151:1261-1303 6194:1304-1313 6204:1314-1399 6289:1400-1409 4316:1410-1633 6515:1634-1655 4727:1656-1688 3752:1689-1807 6683:1808-1990 6863:1991-1994 4905:1995-2020 6892:2021-2042 6914:2043-2097 6967:2098-2102 11083:2103-2272 7142:2273-2279 7149:2280-2491 7354:2492-2504 3672:2505-2793 11268:2794-2795 11270:2796-2815 11290:2816-2822 11297:2823-2833 7691:2834-2850 11309:2851-2899 9829:2900-2906 7764:2907-3004]
TACTAATTCATATGGTGAGTAATGACGAGATAAAGAGCCGTCCGTGTATATTTCACAGTGTTTTTGCAAA
TTCAACTCGGAGAATCCATAGTGTTTAGTTTAGAGAAGTTTCTTGACAGAGTCACTCAGCAGGAGTCCCT
GTTTGCATTGCAATTCCCCTAAAAACAGTGTTTCTCTTTCTCGTTCTCATCCCGTGAACTTCCTGGCCAA
TCCACAATGTCCATAATTCTCGATCTGGACAGCCACAGACGCGAAGTGGTGTCAGATAACAGCGGTTTCT
GCGGCACCCCATCGCCCCATTCCAACTCCTCCACCAGCGACGGCAGCGACGCCACATTCTACGCCCAGGG
AAGCGGTCTGGGACAGCTGTCCTACGACATGAATCAGATGCTGCACTTGCAGGCGTCCCAGGCGCCCTTC
CTGCGGATCATCCATGAGCCTATGGATAAGTTCCGCTTCCGCTACAAGTCAGAGATGCACGGCAGTCACG
GATCCCTGATGGCGGCCACTCCGGACAAGTCCAAGAAGCTCTATCCGACAGTTGAGTTGCGAAACTACAA
CGGAGAGGCAATCATCCGATGTTCTCTCTATCAAACGGACACTGATGGCCCCGATGGCCCAATGCCTCAC
TCCCACAAGCTGGTTGTTCGAACTGGCGATGAGGATCGCGAGGATCCTCACGATGTCATCGTCTCCCCGA
CCGGCTATCACGCCGAGTTCCGCGGAATGGGGATCATTCACACCGCGAAGAAGAACATTGCTGAGGAGCT
TTACCGGAAGCTGAAGACATGCAAAGAGGCGGAACGCGGAGATGTTCTGTCTCTGCGTGATGATGAGAAA
CTGAAGAGTCAGGCAGCCAAGGAGGAGAAAAACATGAACTTGAATCAAGTCTGTCTGGGCTTCCAGTGCT
TCGTGAAGGATCACACGGGGCAATTGAAGAAGTTGTGTGAGCCAGTCTTTTCGAAGCCCATCAATAACAT
GAAAAGCGCCATGACGGGTGAACTGAAGATCGCGAGGATGAGCTGTGCTGTCAGCAGCGTCACGGGGGGC
GAAGATGTCTTCCTGTTTGTGGAGAAAGTCAACAAGAAGAACATCAAGGTGAGATTCTTCGAAGAGGATG
ATCACGGTAACACCGTGTGGCACGCTTATGGGAACTTCACTGAGATTGACGTTCACCATCAATACGGAAT
CGCCCTGACAACGCCCGAGTATCACAGGAAGGACATTAGGACCCAAGTGGACGTCAAGATTCAGCTCGAG
AGACCAGGAGATGGTGAACGGAGCGAGCCTCTGCTTTTCACCTACAAACCTTGTCTGGACGTCATGGCGC
CACGAAAGCGTCCCCGACCCAATGAGAGCTTCAATTCAACGGACATCCCGGCGACGCTCAATCAACTCGA
CCAATTCCCGAGAATCAGTACTGAGTACAACACGAATGAACAGCTGGAGCAACTGTTGCATAATGCCGAT
CAAAATAATCTTCAGGAATTGCAACTTTCAGAGGAGTTTATGTCGCACTTCGCAAAGAATTCTGACGAAT
ACAAAAACATGCTGGACATTAATATGCTGGACCTGACCATGCCGGAATCTGTTCTCTGTCGCGACTCTCC
GGCAAGTTCTGCTCCGGCTGCGGCTGCGGCGGCGAAGAAGGAGTCGACAATGAGCAGTGCTTGTGGTGTT
CCGAGGAACAATGAGTCGATCTATGAGAAGATTCTCTCCATCAATCGCATGTACGGAGTATCGATGCCGA
TCAAAGTGGAGAACATCATCAATCACCTCCTGACGGAGTCCAAGAAGACAGGAGAAAATTTGCTCCATTG
GGCGATTCGAGAGAACGGCGGTCGAGTGAAGAACATCCTTGAGATTGTTCACAAGTTCAATCTCAATGAG
TATCTGGATGAGACGAATGAGAAGGGTGAGAACTGCCTTCATCTCGCTTGTGCTCTGGACAAGGCGGAAT
TCATTCGGCCATTGATCAATTTGGGCGCCAACCCTAATGCGCGCGACATAAACGGAAACACACCGTTGCA
TGTCGCAGTTTTGGAGGGACGCCGGATGTGCCTGGCGAGGATTCTGGATCGCGCCAACTATACGTCCAGG
AGTAAGAGTTTGGACATCAATTTGTCCAATCATCAGAGCATGACGGCTCTCCATCTCGCCGTGAAGAATC
ACGATCTCGACGCTGTGGAGAAACTCGTTAAGGCGGGAGCGTCAGTCAAAATGGCGGAATACAAACATGG
CAACAGTATTTTGCATATTGCCGTTGCCGAACGTGCTGTAGAGGTTGTGAGGTTTTTGCTGCAGGAAACT
GGCGTGAATGTCAATCAGACCAACTCATCCGGTTATACGGCGCTCCAGATGGCTTGCGCCAGCACTGAGA
GTCCGGACTCGAAGGAGATCGTGAGACTTTTGCTGGAACACAAAGCTGATCCGATGATGGTTAACGAGAG
CTTCGTATATATCCCCCAGCCCGCCGATGTCACGCTCAATGAGGACGAGGCGGAGATGGATAGAGCGAGA
AATTCATTTGAATTGGCTCAAAACAAACCCGAAATCTTAGCTGTTCTCCAAAAATTCCACAATTTAGGTC
GGGTGGAGGTGAAGCAGGAAGTCGTTGAGGTGGAGACGGAAGACAAAAATTCCCTGGTGGATGAACTATG
TTTGGGAAAATTGTGCGAAATCTTCAATTCGAATGGCTTGTGGAAGCAACTGGCCATCATTCTGGATCAA
TCGTCTCAGATTTCCATCTGTGAGAGATCATCCAATCCCACCAGGACGCTCTTCAATGCAATCGAGATGC
AAGGAAGTAGCCTGGATGATCTGCTGAAGGCCTGCAACTACCTCAAAATTCCCCAAGCTGTGACCTGCGT
CCAGGAGTTGATCGATAGAAACTTACAAAAGGATGTTAAGCCACCATATCCTTAATTTTGTCTGTTTTCT
TTTTTTATTTTTACATTATGGATAAATTTATATAGGAAATAAATAAGGTACTTAAACTAAAAAAA

**STAT**

>c25329_g2_i3 len=3466 path=[905:0-184 @474@!:185-2190 3087:2191-2203 346:2204-2502 8656:2503-2503 8657:2504-2505 7246:2506-2844 7222:2845-2886 8767:2887-2887 8768:2888-2893 7626:2894-2905 8775:2906-2925 7656:2926-2927 713:2928-3011 832:3012-3465]

AGACGATCCTATATTTTGTCCACTGACACTGTTTATAGGGTCCCCACTCCGCCATTCTACACGCAAGAAG

CAAGTGCGACATGAAGTGAGTCGCTGGATTTTTGTGGTTTTCCATGTGAAATTGTGCGAATAATTGATCA

AAAGTGCGTGAAAACAACACCCAGAGGACTATAAAGTGCAAAGTTAAAAAAAACAACAAGAAACCACGGA

AAGGAGGAAGAGAAGAAGAACGATCGTCAATTTGAGGTGAAGTAGCACAAACCGAGAAGAAAAGATTTCA

AAGAATTTCGACTAAGTGAGAGTGAAGGAAAGAGAGAAAAATGGCTCTTTGGGGGCAAACAGTGAAATTG

CCTGGCGAAGTGATGATGACCATCAAGCAGCACTACAATGAGGTGATGCCGATTGAAATACGTCACTTTT

TAGCGCCATGGCTCGAGGATCATATCCTGCCAACGATGCAGGCTAACGTCCAGATGGATTACGATCCACA

ACATTATGAACAATTGGCATCGAATTTTCTGAGTGACTTTATTTTGGAGTTGGAGACACGGGCGCCTGAT

TTTACGGGGACAAATGTGACGGTCCAGTTGTGCTTCACGGAGATGGCGAAGAAGCTGCGACAGCGATTTC

AGCATGATCCAACACAATTGTTTAGCCACCTGACGCGTTGTTTGCAGATGGAGTGGAACTTTGTGAATGG

GTGTGCGATGCCATATCAGAATGCAGAGATTGTGGAGGTGAATGAGAAGCTTCACACCCTCACCACAATG

GTGGCGGCCAATGAGGAGGAGAATAGACAATTGAAGTTGGATTATGAGCGCTTCTCGTTGGAATATCATG

ATCTTATCAAGACCACCACGTATGTGAAGACAATCACAAATCCGCAGGAATTGGAGCAGAATAAATTGCA

ATTGGGTGAGCAGCAGAAGAGGCTGAATGACTTCCTCAATGCCATCCAGAATCGACAGTTGACACTGATT

GGGAAGATTAAGGAGGTGATTGATCAGATGCAACATGCTCATGAGTTGATTATGGTGAAGCAACTGAGAT

TGTGGAAGGCGAATCAGGGTTTGGCGGGCAATGGGGAGCCGCTGATCAATAATTTGGACACTATTCAGCA

ATGGTGTGAAACATTGGCGGACAATTTGTGGAATACAAAGACACAAATTCAGATCCTGTCGAAAACCAGA

CAGACAATCAATGTACCGATTGATCCCAACTTGCCGGATTTGTTGGAGCCATCTAAGAAAGAGATTATTA

ATTTGCTCGTGAAACTCGTGACAGCCAGTTTTGTCATTGAGAAGCAACCGCCGCAAGTTATGAAGACTAA

TACCAGATTTACAGCCACTGTGCGACTCCTCGTCGGTCCCAAGCTCAACATCCAGATGAATAGTCCCAGT

GTGAGAGTGTCCATTGTATCAGAAACCCAAGCGCAAAATATCCAGCAGACAAATCAATACTCCGAAGCAC

TTGCCTCGGGTGAGATTCTCAATAACAGCGGCACCCTGGAGTACCAACAGGCGACCAATCAGGTGTCGGT

GAGCTTTCGCAATATGCAGCTGAAGAAGATCAAGAGGGCAGAAAAGAAGGGCACAGAGAGTGTTATGGAT

GAGAAGTTTGCCCTCCTCTTTCAGTCAAAATTTCAAATTGGCAGCGGTGAATTGGTTATCACAGCGTGGG

CTCTATCTTTGCCAGTGGTTGTGATTGTTCATGGGAATCAGGAGCCACAGAGTTGGGCCACAATCACATG

GGATAATGCCTTTGCTGATCCGACACGTATACCATTTCAAGTGCCGGACAAGGTGCCATGGCCACGACTC

GCGACAGCGCTCAATACTAAATTCAAAGCGGCCACCGGAAGGGGATTAACCGATCAGAATATGCAATTTC

TCTGTGAGAAGGCTTTCAGGCATGGCTCACCGGGTGCCCTGAATCAGAATGAGATGTACATCTCTTGGGC

GCAATTCTGCAAAGAACCATTACCGGATAGAACATATACGTTCTGGGAATGGTTCTATGCTGTGATGAAG

CTGACTAGGGAGCACTTGCGAAATCCATGGCAGGATGGTCTAATAATTGGATTCATTCACAAGAAACAGG

CAGAGGATATGCTCATGAAGTGTCCGATTGGAACATTTTTGCTTAGATTCTCCGACAGTGAATTGGGTGG

TGTAACAATTGCATGGGTGGGCGAAGGAAGTGACCAGCAACCGCAAATTTTCATGCTTCAGCCATTCTTG

GCAAAGGACTTCTCCATCCACTCCCTCCCTGACCACATTGCTGATCTGCCACAACTTGTCACTCTTCACC

TGGATGTGCCAAAAGAGAGAGCTTTTGGCAAATATTTCACCGCATTTCCAAGTCAAGCGACTAATGGTTA

TGTGAGGCGACGACTGGTGACTCAGGTTCCTGATGTACCTATGGGAAGTTATCCAAACACCCCAAGCCAC

AGCATGAGCATACAATCGCCGGATCACACGAGGGATACACCTTCAATTTCAAGCAGGTACTTGACTCAAT

TCTAAACATTTTTTTCCTGTCTGCCTCTAATTCGCATTAAATTTGAGTTTAATCTTGACAAGAAAAAACA

CAATATCGCACAGCTGCTTCTGTTCACACTAATAATTCATTCATCACAATTCATTTTTCATCCACCCACT

CATACATTTTTCATCTCATCATTCTTAAACGATTCCCAAAACTCATATTCAGTTCTTTATTGGCTTACTT

AGCATTATTCCCCATTTTGAGGCTGTGTATAAATGAAGGAAAAAACATTTTTTTGTTTTCCAACTTAACA

CAACTAAAACCTCATTTATCCATTTTATCATACTAATCCTATTTCCAGCTATACGGGAAGTACATTAATG

CAACTCTAGGCCAATTGAAAAAAAAATATCATAACAAAAATTCATTAACCACGGTTTGTGAGTGAATCTA

ACTTGAGACACTAAAAAAAATCAACTTGCATCAAAAACATTCACCTGTGAGAATGGTAAAAGAAATTTAC

GTAAAAAAATGTCAATCGAGCGATGTGAAGAGAGGCATACCTAATAATCCGCAAGTATTAATCATTGAAT

GATTCTAGAAAATGTATATGGGAAAGTATTTCAATAATTGAGAATTTAGGTATATAGGAAAAAAAATCTG

ACTTATTCTACGATTAGAATACAATTTAAAAAGAAAAAAAAAACTGAGAACAACGTATTGAATGGAGAAA

AAATCGATTGTTTGTTGTGTATTGAATGTTTTATCCTCCAATTTTATTTGGACTAAAAAGAAAGACTTAA

ATAATATCTCTATTAGTGAATGTTAATTGTGTTGTTAAAACTATCATATATTTCTCATTTCTTCTCAAAT

CTATCTGTGGTTGATAAAATCAATTAAACGAAAGTATTATACAAAAAGAAAGATGTTTTAAACTTAAAAT

GTGATGTTGAAAGATAAATTTAACGTGATTGAGCAT

**Dorsal**

>c23272_g1_i3 len=608 path=[2413:0-238 2652:239-351 14678:352-352 14679:353-375 9840:376-389 9854:390-607]
GTCGCCAAGGAGGACATCCAGGTGCGCTTCTACGAGGAGAAGGACGCCCATGTGGTGTGGGAGGCGTTTG
GGGACTTTCAACATACTAATGTACACAAGCAAGTGGCGATAGCCTTCCGGTCGCCCCGATATCGAACACT
CGAAGTGGATCAAATAGTAAAGGCATTTATCCAACTGAAGCGACCATCCGATGGAGCAACGAGTGATCCT
CTGCCATTCGACTACGTACCCCTGGATTCAGTACTTAAACATAAGCGACCAAAAATTGAGAACAGGGACA
TGCTATACAAAAGAACACAAGAACACAATTTGTCCAGGAATCCCCAGCAAACCCTCAATAAACCGGACGG
TAGTAAGTGTTTTTTTTTCTTTTTTTTATCATCAACTCTTTTTTGAATTGTGGGGGTGTTTCTGTCCCAT
TTTCTCACAACATTTCTTCTCTTTGGTGGTGGACTCAATTGTTATTACATTATTACATTATTTATCTGGT
GATGGGATTTTGGGATCATGTCACGATAGAATCTATTTCGCTCCTTCTTTCGCAGATGGATGAATCTCTT
TTTTGTCACGTATTTTATAGTGCATTTTTCGCCCTCGTTGTAACTTGA
