# Supplementary material for: Ecdysone signaling acts as a regulator of immunity and microbiota balance, shaping Leishmania infantum infection in Phlebotomus perniciosus
Source: Front Immunol. 2026 Aug 11;17:1904500. doi: 10.3389/fimmu.2026.1904500 (PMC13503605; doi:10.3389/fimmu.2026.1904500)
Supplement: Supplementary file 4 [file Table4.docx]

**Additional file 4. Primer amplification efficiencies used for RT-qPCR analyses**

| **Species** | **Target** | **Slope** | **Amplification factor (E)** | **Efficiency (%)** | **Source** |
| --- | --- | --- | --- | --- | --- |
| *P. perniciosus* | serpent | 1.000 | 2.0000 | 100.00 | Present study |
| *P. perniciosus* | STAT | 1.050 | 1.9351 | 93.51 | Present study |
| *P. perniciosus* | relish | 1.006 | 1.9917 | 99.17 | Present study |
| *P. perniciosus* | dorsal | 1.200 | 1.7818 | 78.18 | Present study |

**Notes**

• dsEcR and dsLacZ correspond to dsRNA/RNAi templates or controls and are not included as RT-qPCR primer-efficiency entries.

• GAPDH, EF-1alpha, EcR, Eip75B, attacin, defensin 1 and defensin 2 were previously reported in Vieira et al. (2024) and/or Sassu et al. (2024), as indicated in the main manuscript primer table.

• *Leishmania* actin and bacterial 16S rRNA primers sequences were provided from Di-Blasi et al. (2015) and Nadkarni et al. (2002), respectively.
